# Attitudes of patients with a rheumatic disease on drug use in the COVID-19 pandemic

**DOI:** 10.1186/s42358-021-00211-6

**Published:** 2021-09-03

**Authors:** Belkıs Nihan Coskun, Burcu Yagiz, Yavuz Pehlivan, Ediz Dalkilic

**Affiliations:** 1 Division of Rheumatology, Uludağ University Faculty of Medicine (Uludağ Üniversitesi Tıp Fakültesi Romatoloji Bilim Dalı), Görükle, Nilüfer, Bursa 16059 Turkey; 2 Division of Rheumatology, Afyonkarahisar State Hospital, Afyon, Turkey

**Keywords:** Anti-rheumatic drugs, Biological treatments, COVID-19, Discontinuation, Rheumatic patients

## Abstract

**Background:**

Anti-rheumatic drugs can increase the predisposition to infection, and patients may be unaware of continuing their treatment during the COVID-19 pandemic.

**Objective:**

This study aimed to assess whether patients maintain their treatment for rheumatic conditions during the pandemic period and determine the factors responsible for discontinuation.

**Methods:**

Patients were randomly selected from the prospectively collected database of our tertiary referral center. The patients were interviewed by telephone through a standardized closed-ended questionnaire, which is targeting the continuity of the treatment plan and the considerations related to the individual choice. The patients were asked whether they hesitated to visit the hospital for follow-up or intravenous drug administration.

**Results:**

A total of 278 patients completed the questionnaire. While 62 of the patients (22.3%) had reduced or interrupted the treatment, only 11 patients (3.9%) stopped the treatment completely. A significant difference was observed between the duration of illness and the discontinuation of treatment. (p = 0.023) There was a significant difference in disease activity between the group that stopped treatment and continued treatment. (p = 0.001) There was no statistically significant difference in other demographic characteristics. One hundred thirty-five patients (48.6%) made the treatment decision by themselves, and 80% continued the treatment. Reasons for stopping the treatment were anxiety (48.4%), not being able to go to the hospital for intravenous treatment (45.1%), and not being able to find the drug (6.5%).

**Conclusion:**

Since patients with long-term illnesses were found to be significantly more likely to stop their treatment, this group of patients should be monitored.

## Introduction

In December 2019, coronavirus disease 2019 (COVID-19), caused by severe acute respiratory coronavirus 2 (SARS-CoV-2), first emerged in Wuhan, China. The virus spread quickly worldwide due to its high disease transmission rate [1, 2]. Since the diagnosis of the first cases, nearly 120 million people were confirmed as positive, and 2.6 million died in the COVID-19 pandemic by the end of March 2021 [3].

While the disease is usually asymptomatic or with minor symptoms in many cases, 15–20% develop interstitial pneumonia and acute respiratory distress syndrome (ARDS) [4]. Several risk factors are responsible for the development of severe symptoms, including older age, comorbidities such as hypertension (HT), diabetes mellitus (DM), obesity, high-dose corticosteroid use, and immunodeficiency [5].

Patients with rheumatic diseases have an inherent risk for bacterial, viral, or opportunistic infections compared to the general population. Also, the use of immunosuppressive medications such as conventional synthetic disease-modifying anti-rheumatic drugs (csDMARDs), biological disease-modifying anti-rheumatic drugs (bDMARDs), and corticosteroids in patients with autoimmune rheumatoid disease, increase the risk of these infections [6, 7].

Uncontrolled disease activity is one of the most sensitive and specific independent infection risk predictors, especially during the pandemic. On the other hand, the immunosuppressive treatment may increase the risk of infection, and therefore, the patients may hesitate to continue their ongoing treatment plan [8, 9].

There is currently a lack of information regarding the effect of SARS-CoV-2 on patients suffering from rheumatoid diseases. However, several international associations of rheumatoid diseases such as the European League against Rheumatology (EULAR), American College of Rheumatology (ACR), British Society for Rheumatology (BSR), and Italian Society for Rheumatology (SIR) recommend continuing immunosuppressive therapies to prevent relapses [10–13]. This advice helps prevent relapses that may occur when therapy is eventually stopped [14].

There is limited data on patients' preferences on the continuation of the treatment during the pandemic phase. Patient surveys from Greece, Italy, and Latin America reported a compliance rate between 85 and 97.8% [14–16]. This study aimed to survey whether the patients diagnosed with rheumatoid arthritis (RA) and ankylosing spondylitis (AS) continue their medications during the pandemic phase and determine the factors responsible for discontinuation.

## Methods

Following the approval by the institutional board review, a telephone survey was conducted between the dates 1/7/2020 and 20/7/2020. The patients were interviewed by telephone through a standardized closed-ended questionnaire.

### Patients characteristics

The patients diagnosed with RA or AS receiving corticosteroids, non-steroidal anti-inflammatory drugs, csDMARDs, bDMARDs and selective targeted synthetic disease-modifying (tsDMARDs) treatments were expected to participate in the study. The 2010 EULAR/ACR guidelines were used to diagnose patients with RA [17]. In diagnosing SpA patients, the modified New York criteria and EULAR criteria for axial and peripheral SPA 2009 were used [18, 19].

Patients were randomly selected from a prospectively collected database of our tertiary referral center. The participation of a total number of 300 patients was targeted. Every 5th patient was chosen for the survey on the database as the randomization procedure. The patients with a follow-up duration longer than 6 months were included in the study. Patients under the age of 18 and who had invalid contact information were excluded.

The demographic information including age, gender, the region of residence (urban, sub-neighborhood, rural), the education level (First, second, third), employment status, smoking, rheumatological diagnosis, disease duration, current treatments, comorbidity (HT, coronary heart disease, DM, chronic obstructive pulmonary disease (COPD)) was obtained from the database.

### Questionnaire features and application method

A single rheumatologist (BNC) interviewed with all of the patients. The patients were asked whether they continued their ongoing treatment plan and how they decided to continue or stop treatment after 15th March 2020 when the first COVID-19 cases were reported in Turkey. The patients were asked if they hesitated to visit the hospital for follow-up or intravenous drug administration. Also, patients were asked whether they referred to a hospital due to COVID-19 symptoms and whether they had a history of hospitalization for an infection in the past year. The perception of disease control was evaluated by using the Visual Analog Scale.

### Statistical analysis

SPSS version 22.0 was used for statistical analysis. Data were presented as the mean and standard deviation or, when applicable, number and percentage. For categorical data comparisons, Fischer's Exact Test was used, and the independent t-test was used to determine parameters when applicable. A p-value of less than 0.05 was considered statistically significant.

## Results

**Fig. 1:**
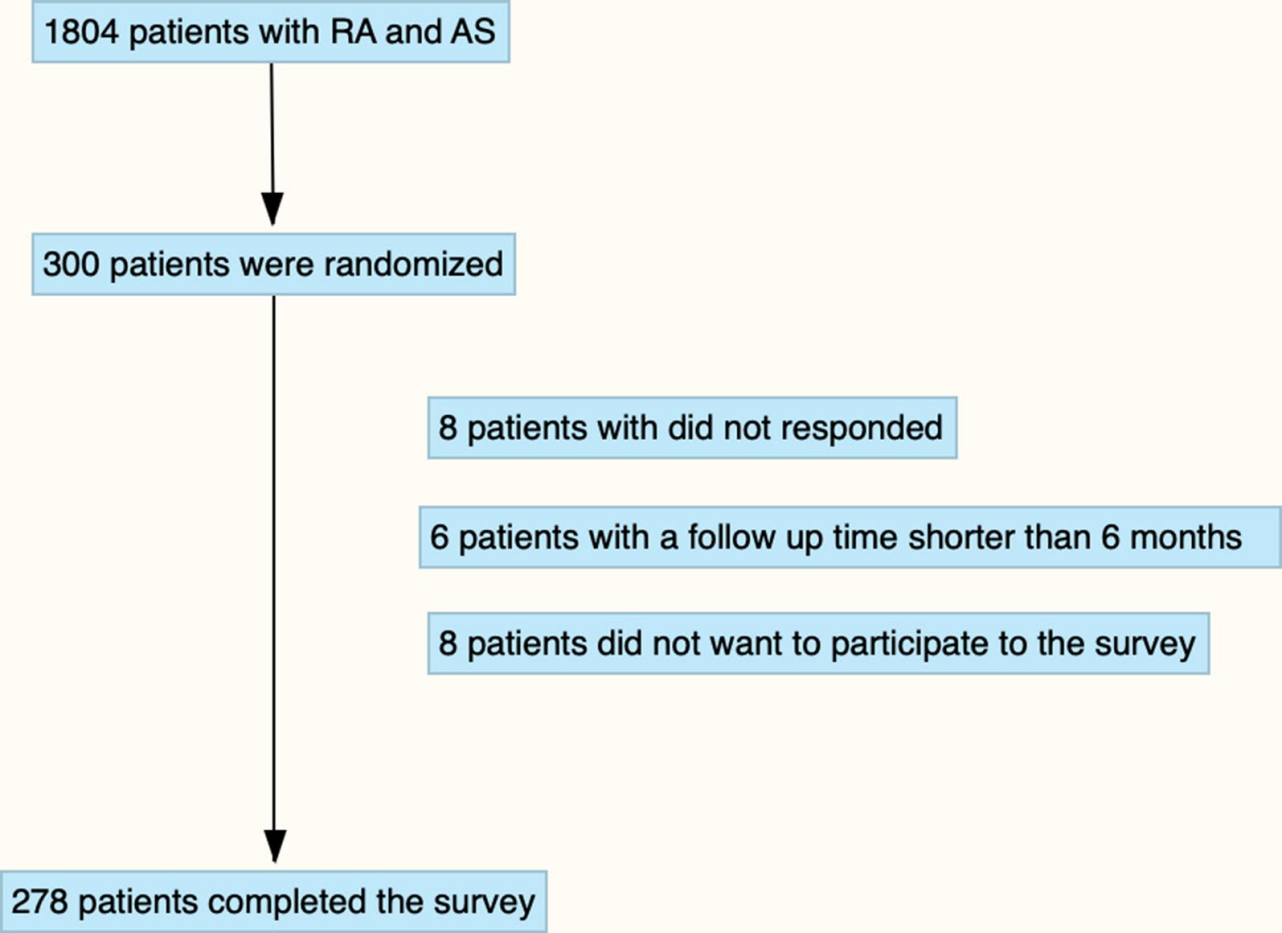
Flowchart of the planning strategy of the article

A total of 278 patients (146 RA, 132 AS) completed the survey as shown in Fig. 1. The mean age of the patients was 47.53 ± 13.02 years (19–78). One hundred seventy-nine patients (64.4%) were female. A total of 62 (%22.3) patients discontinued or interrupted their ongoing treatment plan. Only 11 patients (3.9%) completely stopped their treatment. A comparison of the demographic variables in patients according to the continuation status was reported in Table 1. While the mean disease duration in patients who continued to their treatment plan was 11.15 ± 8 years, and the mean disease duration in patients who interrupted/stop their treatment plan was 13.93 ± 9.79 years. Disease duration was significantly higher as a contributing factor (p = 0.023). A significant difference was observed between the duration of illness and the discontinuation of treatment. There was a significant difference in disease activity between the group that stopped treatment and continued treatment. (p = 0.001) There was no statistically significant difference in other demographic characteristics. Table 2 listed the rate of drugs in patients with their continuous status. The continuation rate of DMARDS, bDMARDs, tsDMARD, csDMARDs was 73.3%, 83.3%, 95.4%, respectively. Low-dose glucocorticoids and NSAIDs were maintained in 95.5% and 96.4% of the patients, respectively.

**Table 1:** Comparison of the demographic variables in patients acoording to the contiuation status

	Ongoing (n = 216)	Interrupted/stop (n = 62)	p
Age	47.75 ± 13.09	46.8 ± 12.86	0.616
Gender			0.073
Female	133 (74.3%)	46 (25.7%)	
Male	83 (83.8%)	16 (16.2%)	
Diagnosis			0.248
Rheumatoid arthritis	109 (74.7%)	37 (25.3%)	
Spondylitis	107 (81.1%)	25 (18.9%)	
BMI	26.9 ± 5.18	28.27 ± 6.39	0.083
Smoking			0.327
Active	58 (78.4%)	16 (21.6%)	
Non-smoker	134 (75.7%)	43 (24.3%)	
Previous	24 (88.9%)	3 (11.1%)	
Disease duration	11.15 ± 8	13.93 ± 9.79	**0.023**
Disease activity (VAS)	7.76 ± 1.7	6.61 ± 2.33	**0.001**
Education			0.079
Low	98 (81%)	23 (19%)	
High school	81 (80.2%)	20 (19.8%)	
University	37 (66.1%)	19 (33.9%)	
Occupation			0.771
Occupant	89 (79.5%)	23 (20.5%)	
Non-working	106 (75.7%)	34 (24.3%)	
Retired	21 (80.8%)	5 (19.2%)	
Mariatial			0.582
Single	21 (75%)	7 (25%)	
Married	187 (78.2%)	52(21.8%)	
Divorced/widowed	6 (66.7%)	3 (33.3%)	
Comorbidity			0.326
Hypertension	34 (70.8%)	14 (29.2%)	
Coroner artery disease	5 (71.4%)	2 (28.6%)	
Diabetes mellitus	14 (82.4%)	3 (17.6%)	
Pulmonery disease	2 (50%)	2 (50%)	

Bold values refer to statistical significance values

**Table 2:** Distribution of ongoing and Interrupted/stop drugs

Drugs	Ongoing	Interrupted/stop
bDMARD	148 (73.3%)	55 (26.7%)
Anti-TNF		
Adalimumab	30 (90.9%)	3 (9.1%)
Certolizumab	17 (85%)	3 (15%)
Etanercept	29 (74.3%)	10 (25.7%)
Golimumab	18 (81.8%)	4 (18.2%)
Infliximab	13 (56.5%)	10 (43.5%)
Tocilizumab	11 (47.8%)	12 (52.2%)
Rituximab	19 (65.5%)	10 (34.5%)
Secukinumab	3 (60%)	2 (40%)
Abatacept	3 (100%)	0 (0%)
tDMARD		
Tofasitinib	5 (83.3%)	1 (16.7%)
csDMARD	124 (95.4%)	6 (4.6%)
Methotrexate	69 (95.8%)	3 (4.2%)
Leflunomide	40 (100%)	0 (0%)
Sulfasalazine	15 (100%)	0 (0%)
Hydroxychloroquine	70 (93.3%)	5 (6.7%)
Low-dose glucocorticoids	84 (95.4%)	3 (4.6%)
NSAIDs	134 (96.4%)	5 (3.6%)

One hundred and thirty-five patients (48.6%) made the treatment decision independently, 80% continued the treatment. One hundred eleven patients (39.9%) made a co-decision with the rheumatologist, and 76.6% maintained the treatment. The other 32 patients (11.5%) decided by consultation with other healthcare professionals such as family physicians, nurses, and pharmacists. Of these cases, 71.9% continued the treatment (p = 0.571). It was found that the decision on the medication was made mainly by contacting the rheumatologist. The reasons for stopping/interrupting medication were anxiety (48.4%), inability to visit the hospital for intravenous treatment (45.1%), and inability to obtain the drug (6.5%). 89.6% of the patients had concerns about visiting the hospital. There were only three patients (1.1%) who had an infection requiring hospitalization last year. In the past 3 months four patients with suspected COVID-19 symptoms such as cough and back pain were admitted to the emergency room. The COVID-19 tests performed on these patients have been reported to be negative.

## Discussion

The inherent risk of infection in rheumatic patients, in addition to the medication's immunosuppressive effects, can lead patients to remain concerned regarding treatment continuity. Any patients who suspected the treatment's immunosuppressive effects stopped or disrupted their current treatment either in compliance with their own decisions or in line with the health authorities' recommendations. Patients may also have trouble reaching physicians for information or cannot access their intravenous treatments when ambulatory health services have been shut down. Since the pandemic has not stopped and a second wave is likely, we aim to examine the actions and behaviors of treatment for patients with rheumatological conditions and explore the factors that will help them prepare more accurately.

Our study found that a great majority of the patients have retained in their current treatment with a continuation rate of 77.6%. In the first three months since the pandemic announcement, approximately one in five patients extended the treatment dose intervals, while only 11 patients stopped their medication completely. Similarly, in a study of 500 patients, Fragoulis et al. reported that 11 patients (2.2%) stopped immunosuppressive drug therapy [15]. Another Latin American study revealed that 90% (n = 293) of patients did not modify their drug schedule [16]. In various studies conducted in Italy, America, Spain and Turkey, it was reported that approximately 75–80% of patients did not change their treatment [20–23].

Following the pandemic's announcement, EULAR, ACR, BSR, SIR, and Turkish Rheumatology Association issued recommendations in March–April that patients with rheumatic disease should not stop medication treatment since any discontinuation in treatment can lead to a relapse of rheumatological diseases and may increase the risk of infection further. Due to the positive effects of both national and international recommendations, drug treatments may have been left to a lesser extent [10–13, 24].

In our research, patients were found to postpone or delay their treatments, most notably because they were fearful of the predisposition to infection induced by their therapies (48.4%). Similarly, fear of contagion COVID-19 infection, which is the most common reason for stopping or delaying treatment, has been reported in other studies with rates as high as 80% [21, 25]. The second most common reason was the lack of service in hospitals that refer to intravenous treatment (45.1%). The discontinuation rate due to the inability to obtain the medication (6.5%) was very low.

Before the pandemic, patients evaluated by rheumatologists in routine practice in our country were able to access biological treatments only after obtaining a committee report and prescription was written. However, after the announcement of the pandemic, patients with a prior committee report had the right to obtain their biological drugs from the pharmacy without seeing the doctor and without a prescription by the permission of Ministry of Health of The Republic of Turkey [26]. This condition may be an essential factor for patients to continue drug treatment.

In the present study, the group that stopped treatment was found to have a longer disease duration than those who did not stop. Patients using long-term immunosuppressive drugs have been shown to have a predisposition to anxiety and behavioral disorders. This situation may explain why discontinuation is high in patients with a long disease duration due to anxiety [27]. No statistically significant difference between the group that stopped the treatment and the group that did not was found when comparing education level, age, profession, comorbidities, and body mass index. Although this result was not statistically significant, we found a high propensity to stop among those with high education. Fragoulis et al. found that the discontinuation rates were associated with specific clinical and social conditions such as unemployment status and COPD [15]. In this study, four patients had pulmonary disease. Two of them continued treatment while two intermittently used it.

Typically, extreme COVID-19 symptoms were associated with obesity, age, comorbidities such as lung disease, DM, history of previous serious infections, long-term corticosteroid therapy, and higher underlying rheumatic disease activity [28, 29]. We observed a trend in obese patients to avoid drug therapy while not statistically significant.

The steroid drugs are among the most essential medicines used in managing rheumatological disorders, in terms of increased risk of infection [30]. Although it is generally known as a tendency to infection, discontinuation of steroids and other immunosuppressive drug treatments has not been recommended by associations such as EULAR, ACR [10, 11]. A recent study also reported the efficacy of low-dose steroid therapy in the treatment of severe COVID-19 infections [31]. Approximately 1/3 of the present study patients were using steroids with a discontinuation rate of 3.4%. In a retrospective analysis published in China, 6 of the 21 rheumatic patients with COVID-19 infection were using glucocorticoid therapy before infection. All of these patients were found to improve. During the follow-up, four patients received steroids due to rheumatologic disease reactivation. A total of 37.5 mg glucocorticoid treatment was administered to 10 patients during the treatment [32]. Continuing the ongoing steroid treatment is necessary to stop reactivation of the condition and to prevent potential infections.

Patients with immunocompromises are at high risk of multiple infectious diseases, including infections with the viruses. TNF-α, IL-6, and IL-1 are crucial pro-inflammatory cytokines in body defense. Blocking these cytokines may cause especially infections with intracellular infectious agents; however, it creates a predisposition to all kinds of infections[33]. Due to the increased risk of infection, the use of bDMARD may be a concern in patients during the COVID-19 period. Although the increased risk of serious infections has been reported in meta-analyses to date in those receiving biological treatments, we do not have clear data on whether this risk increases COVID-19 infection [10].

In the present study, the bDMARD usage rate was 73.3%, with a discontinuation rate of 27%. Although there are no clinical studies on whether these drugs negatively affect the course of COVID-19 infection in patients using anti-TNF agents, few case reports are available in the literature. There is no proven data to show that these drugs have positive or negative effects [34–36].

Tocilizumab (TCZ), a monoclonal antibody against interleukin‐6 (IL‐6), has recently emerged as an alternative therapy for COVID‐19 patients at risk for cytokine storms. Which inflammatory cytokine is most important in the pathogenesis of COVID-19 is currently unknown, although IL-6 appears to be crucial [37]. The cytokine storm causing ARDS because of excessive immune reaction may be fatal in COVID-19 infection. Therefore, anti-cytokine intervention may not affect the risk of viral infection and viral clearance but may inhibit the hyperinflammatory state in COVID-19, which can be beneficial [37]. No data has been shown that it is protective against COVID-19 infection in TCZ users due to rheumatic disease. 52.5% of our patients had interrupted TCZ treatment. All of those who interrupted were receiving TCZ treatment intravenously. The interruption was due to the inability to reach the hospital, but all patients continued their medication, albeit with a delay.

Hydroxychloroquine (HCQ), an anti-malarial drug, acts by increasing endosomal PH. It inhibits immune activation by preventing signaling and cytokine production through Toll-like Receptors (TLR) at the cellular level. It has an inhibitory effect on viral replication in ex vivo, but this has never been demonstrated in randomized controlled trials [38].

There is no data supporting the use of HCQ as a preservative, and it is not included in the guidelines. In Italy, 4 of 320 chronic rheumatology patients were found to be Covid-19 positive, and these four patients were infected while using HCQ. None of these patients with a mean age of 58 ± 5 years, who also used biological therapy, had a severe respiratory syndrome or death [39, 40].

Only 6.7% of our patients had interrupted the treatment due to the inability to obtain the drug. Although its effectiveness in prophylaxis has not been demonstrated, learning the use of HCQ in the treatment of COVID-19 may have aroused more interest in the drug in patients. Effective and acceptable use of HCQ in the treatment of SARS-CoV-2 may be one factor that has increased drug retention.

Vitamin D modulates the innate and adaptive immune system, and its deficiency has been understandably linked to an increase in autoimmunity and infection predisposition [41]. Grant et al. (2020) pointed to the importance of vitamin D in reducing the incidence of COVID-19 infections in the respiratory tract [42]. The rate of using vitamin D supplements in our patients 21.2%. Vitamin C is one of the critical components that makes a strong immune system of water-soluble vitamins. The specific types of products and food must be known in the current situation for combating COVID-19 to improve our immune system [43]. The rate of using vitamin C supplements in our patients was 5.4%.

At the beginning of the epidemic, tight closure policies were implemented in most countries of the world. In the first three months of the epidemic in Turkey, there was a period of strict isolation, especially for people over the age of 65 years and under the age of 18 years.

In the same period, people with chronic diseases were deemed on administrative leave, many workplaces switched to online working order and many patients were self-quarantined. In a period when such strict measures are taken, it seems possible that rheumatism patients will make changes such as the anxiety of getting COVID 19 infection and reduction in drug treatment. There may also be an increased interest in supplements to protect patients from infection.

The limited number of patients is the main limitation of the current study. In this study, although it was theoretically possible to reach a higher number by sending e-mails to all patients in the database, only telephone access was selected to analyze the data in better quality due to the consideration of the socio-cultural status and low education level of the patients.

## Conclusions

The adherence of rheumatology patients to treatment was investigated in this study in the early period after the announcement of the COVID-19 pandemic, and it was found that treatment adherence was high. It was determined that the most important factor in the discontinuation of the drug was the concern of infection with COVID 19. It was observed that treatment was considerably more stopped, particularly in patients with longer illness duration. Therefore, it may be intended to provide patients with better information not to disrupt therapy.

## Declarations

### Availability of data and materials

During the current study, the datasets used and analysed are available from on a reasonable request by the corresponding author.
